# Comparative Efficacy of Traditional Corticotomy and Flapless Piezotomy in Facilitating Orthodontic Tooth Movement: A Systematic Review and Meta-Analysis

**DOI:** 10.3390/medicina59101804

**Published:** 2023-10-10

**Authors:** Sung-Hoon Han, Won-Jong Park, Jun-Beom Park

**Affiliations:** 1 Department of Orthodontics, Seoul Saint Mary’s Hospital, College of Medicine, The Catholic University of Korea, Seoul 06591, Republic of Korea; scherazade@hanmail.net; 2 Department of Oral and Maxillofacial Surgery, Seoul St. Mary’s Hospital, College of Medicine, The Catholic University of Korea, Seoul 06591, Republic of Korea; roll8888@naver.com; 3 Department of Periodontics, College of Medicine, The Catholic University of Korea, Seoul 06591, Republic of Korea; 4 Dental Implantology, Graduate School of Clinical Dental Science, The Catholic University of Korea, Seoul 06591, Republic of Korea; 5 Department of Medicine, Graduate School, The Catholic University of Korea, Seoul 06591, Republic of Korea

**Keywords:** orthodontics, piezosurgery, surgical procedures, operative, tooth movement techniques

## Abstract

*Background and Objectives*: This study aimed to perform a meta-analysis comparing the effects of corticotomy and flapless piezocision on accelerated tooth movement. *Materials and Methods*: A comprehensive search using a combination of controlled vocabulary (MeSH) and free-text terms was undertaken by two reviewers to identify published systematic reviews. Three major electronic databases (Medline via PubMed, Cochrane Database, and Embase) were searched up to 2 June 2023. *Results*: The results of the meta-analysis showed that the pooled standardized mean difference values of accumulative movement distances for flapless piezocision were 1.43 (95% CI, 0.38 to 2.48; *p* < 0.01), 1.09 (95% CI, −0.08 to 2.26; *p* = 0.07), and 0.73 (95% CI, −0.58 to 4.02; *p* = 0.14). The results of the meta-analysis demonstrated that the pooled SMD values of accumulative movement distances for the corticotomy were 2.76 (95% CI, 0.18 to 5.34; *p* = 0.04), 1.43 (95% CI, −1.10 to 3.96; *p* = 0.27), and 4.78 (95% CI, −4.54 to 14.10; *p* = 0.32). Although the test for overall effectiveness was significant for piezocision and corticotomy, there were no significant differences between piezocision and corticotomy. *Conclusions*: The study determined that both conventional corticotomy and flapless piezosurgery are effective as adjuncts to orthodontic treatment. Moreover, no significant difference was observed in the short-term effectiveness of canine retraction acceleration between conventional corticotomy and flapless piezocision. While piezocision may be a favorable option for orthodontic treatment, corticotomy can be considered in cases requiring additional procedures such as bone grafting.

## 1. Introduction

Traditional orthodontic treatment frequently requires long treatment periods, which can result in higher patient dissatisfaction and elevated treatment expenses [1,2]. Prolonging the treatment duration by 6 months can result in a significant 23% reduction in patient adherence [3]. In addition, the risk of iatrogenic complications, including root resorption, may increase as the duration of treatment increases [4]. In an attempt to tackle these challenges, dental professionals have been exploring diverse methods to expedite tooth movement [5,6,7,8,9,10]. Corticotomy was introduced as a supplementary approach to conventional orthodontic treatment and includes a combination of inter-radicular osteotomy and elevation of a mucoperiosteal flap [11]. In 1959, Köle introduced corticotomy to facilitate rapid tooth movement by surgically cutting the alveolar bone [12,13]. He applied corticotomy and osteotomy to address different types of malocclusion. Moreover, a surgical method utilizing corticotomy to facilitate en-bloc relocation of teeth within the bone has been employed in orthodontic treatment with the aim of decreasing treatment duration and minimizing root resorption [12,14]. Subsequently, this concept was modified by Wilcko et al., who suggested that tooth movement is facilitated by the demineralization/remineralization process. They proposed a revised protocol that encompasses both buccal and lingual full-thickness flaps, selective partial decortication of the cortical plates, simultaneous bone grafting or augmentation, and the closure of the primary flap [15]. A previous report revealed a significant increase in the rate of tooth movement compared to conventional orthodontic treatment, demonstrating an approximately 2.5-fold difference during the short-term period following corticotomy [16,17,18].

Although the previous report indicated that corticotomy can enhance the pace of tooth movement, its invasiveness can often dissuade patients due to discomfort and potential complications [19]. Accordingly, piezocision, a less invasive surgical intervention, has been introduced as an alternative to conventional corticotomy [20]. Piezocision uses an ultrasonic microsaw to create small, controlled incisions without raising a full-thickness flap, which allows for precise cutting of the bone around the tooth [21]. Although studies investigating the effects of this dental approach have been conducted, the results remain inconsistent and relatively limited [22,23,24]. Several studies have documented noteworthy acceleration in the pace of tooth movement when comparing piezocision to traditional orthodontic approaches [18,25]. Conversely, several studies have indicated no significant distinctions in the results between piezocision and conventional orthodontic treatment [24,26].

Therefore, the effects of flapless piezocision on accelerated tooth movement have been a subject of ongoing debate. Despite its advantage of being less invasive compared to corticotomy, piezocision remains an uncertainty in the clinician’s options. Therefore, this study aims to perform a meta-analysis comparing the effects of conventional corticotomy and flapless piezocision on accelerated tooth movement. The null hypothesis was that there was no significant difference in the acceleration rate of canine retraction between corticotomy and piezocision.

## 2. Materials and Methods

### 2.1. Protocol and Registration

**Figure 1:**
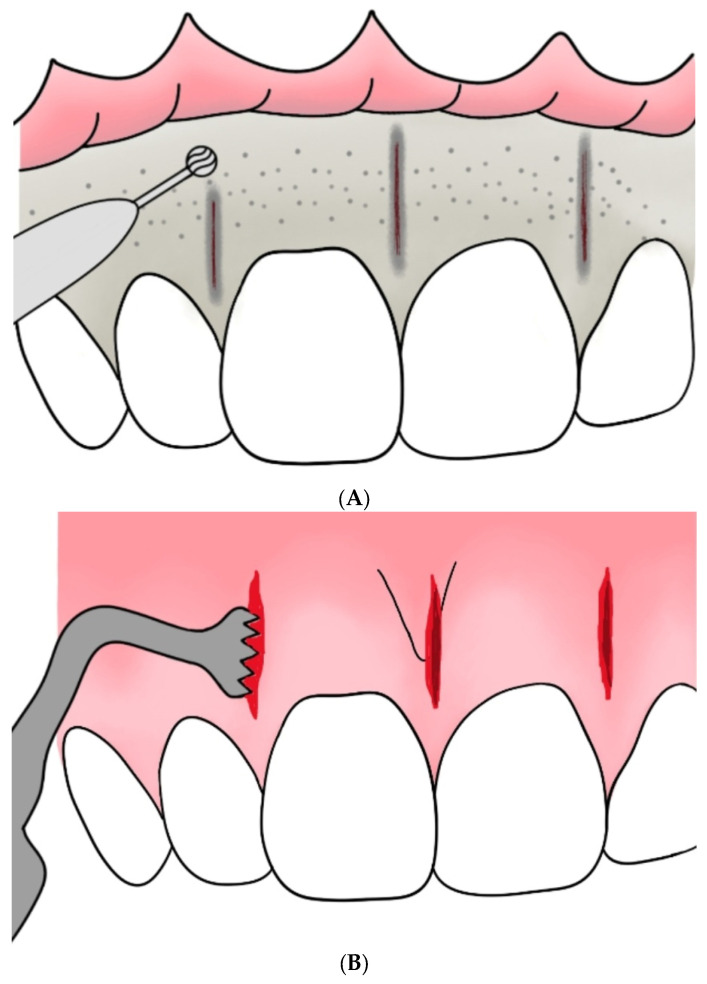
Schematic representations. (**A**) Visual representation of corticotomy. (**B**) Schematic drawing of flapless piezoincision.

Figure 1 illustrates schematic representations of both methods: corticotomy and flapless piezoincision. The recommendations of the Preferred Reporting Items for Systematic Reviews and Meta-Analysis (PRISMA) Statement are followed in the systematic review [27].

### 2.2. Eligibility Criteria

Question: Is flapless piezocision more effective than corticotomy in accelerating orthodontic tooth movement?

Participants: Orthodontic patients with upper first premolar extraction.

Interventions: Canine retraction with flapless piezocision or corticotomy.

Comparisons: Canine retraction with conventional fixed orthodontic treatment only.

Outcomes: Canine retraction rate (mm/month) after 1, 2, and 3 months from piezocision or corticotomy.

Study design: Randomized controlled trials (RCTs).

We excluded in vitro and animal studies, studies with sample sizes smaller than six, studies referring to accelerated tooth movement occurring as a result of orthognathic surgery, distraction osteogenesis, surgical acceleration intervention for palatal expansion, and non-surgical acceleration procedures, pharmacological approaches, and studies that did not involve extraction of maxillary first premolars. Moreover, literature reviews, case reports, case-control studies, retrospective study designs, and studies published in languages other than English were excluded.

### 2.3. Information Sources and Search Strategy

A comprehensive search using a combination of controlled vocabulary (MeSH) and free-text terms was undertaken by two reviewers (SHH and WJP) to identify published systematic reviews. Three major electronic databases (Medline via PubMed, Cochrane Database, and Embase) were searched up to 2 June 2023. A manual search was also carried out in the four leading orthodontic journals (American Journal of Orthodontic and Dentofacial Orthopedics, European Journal of Orthodontics, Journal of Orthodontics, and Journal of Orthodontics and Angle Orthodontists). Grey literature searching for unpublished data was carried out on the ProQuest (https://www.proquest.com accessed on 2 June 2023) and OpenGrey Europe (http://www.opengrey.eu accessed on 2 June 2023) websites. The search results were exported to EndNote reference management software (Version 21, Clarivate, Philadelphia, PA, USA) for removal of duplicates. The established procedure entailed reaching out to the lead authors of chosen studies on two consecutive weeks through email, either to elucidate specific study information or to request raw data if necessary. The search approach was adapted based on the specific requirements of each database. Details on the search strategy are presented in Supplementary Tables S1–S3.

### 2.4. Study Selection and Data Extraction

The title and abstract of the retrieved papers were screened by two reviewers (SHH and WJP) blinded to the eligibility criteria. Any disagreement was resolved by discussion with another author (JBP). Finally, full text of the remaining articles was reviewed independently and in duplicate by the two reviewers for final inclusion. Inter-reviewer agreement was tested using the Kappa test. Data were extracted independently from the included studies according to the PICOS question and arranged in topics of general information (author name and publication date), participant variables (size, age, sex, and main inclusion criteria), intervention/comparison (flapless piezocision/conventional corticotomy), outcomes (canine retraction ratio after 1, 2, and 3 months from surgical intervention), and study design.

### 2.5. Risk of Bias Assessment

The reviewers used the Cochrane Risk-Of-Bias (ROB 2.0) tool for randomized studies. The guideline checklist included questions on the randomization process (selection bias), deviations from the intended interventions (performance bias), missing outcome data (attrition bias), measurement of the outcome (detection bias), selection of the reported result (reporting bias), and overall bias. The risk of bias of the recruited studies was evaluated as low risk, some concerns, or high risk. The quality of the eligible studies was evaluated by the two reviewers. 

### 2.6. Data Synthesis and Analysis

Meta-analysis was performed using R (version 3.5.0; R Project for Statistical Computing). The mean difference (MD) and 95% confidence interval (CI) were used as summary statistics. A random-effects method was used for meta-analysis. The level of significance was set at 0.05. I^2^ and chi-square tests were performed to quantify the heterogeneity across studies.

## 3. Results

### 3.1. Study Selection

**Figure 2:**
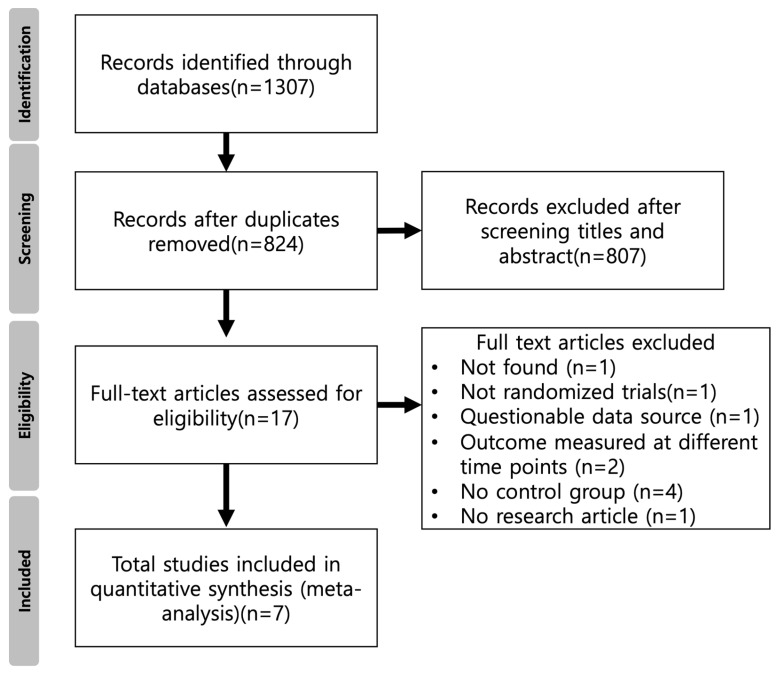
Flow chart of the included studies.

The initial search identified 1307 articles. After 483 duplicates were excluded, the article titles and abstracts were read, and 807 articles were removed due to failure to meet the inclusion criteria. The inclusion and exclusion criteria were applied to the remaining 17 full-text articles. After 10 articles that did not meet the inclusion criteria were excluded, 7 studies were assessed for eligibility. The literature-screening flow chart is shown in Figure 2.

### 3.2. Risk of Bias Assessment

**Figure 3:**
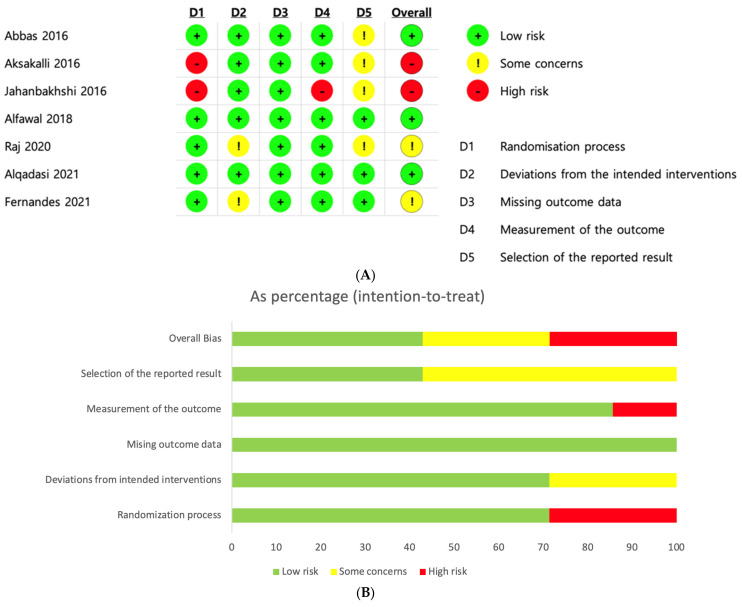
Risk of bias. (**A**) Summary of the risk of bias in the included studies [18,28,29,30,31,32,33]. (**B**) Overall risk of bias score for each process step.

The summary of the risk of bias and overall risk of bias score for each step in the included studies are shown in Figure 3. In all included articles, the effect of blinding of patients and personnel was not possible. However, the blinding of the outcome assessment was critical and considered to affect the outcome. Overall, three trials were defined as having a low risk of bias, two were considered to have a high risk, and two had some concerns about biases. The major reasons why the two trials exhibited some concerns about biases resulted from unclear deviations from the intended interventions and selection of the reported result. High risk of bias in two studies was due to an inadequate randomization process, measurement outcome, and selection of the reported result.

### 3.3. Meta-Analysis

Table 1 provides an overview of the key attributes of the studies incorporated in this analysis. Considering the *I*^2^ values and the results of the sensitivity analysis, which indicated significant heterogeneity among the studies, a random effects model was applied.

**Figure 4:**
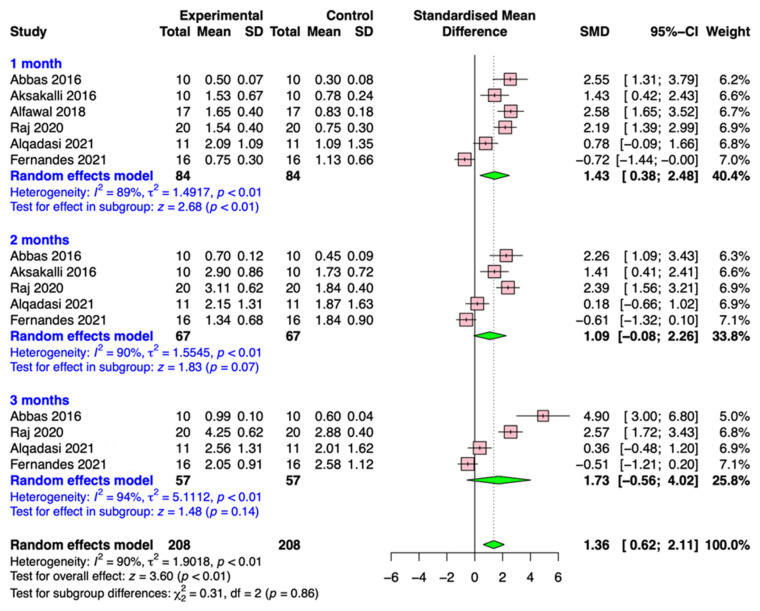
Forest plot of the meta-analysis on studies involving piezocision [18,28,29,31,32,33].

Six included articles (Abbas et al., 2016 [28]; Aksakalli et al., 2016 [29]; Alfawal et al., 2018 [18]; Raj et al., 2020 [31]; Alqadasi et al., 2021 [32]; Fernandes et al., 2021 [33]) assessed the effect of flapless piezocision on canine retraction; the results are presented as forest plots in Figure 4. The results of the meta-analysis demonstrated that the pooled SMD values of accumulative movement distances for flapless piezocision were 1.43 (95% CI, 0.38 to 2.48; *p* < 0.01), 1.09 (95% CI, −0.08 to 2.26; *p* = 0.07), and 0.73 (95% CI, −0.58 to 4.02; *p* = 0.14). Although the overall effect of the procedures was significant (*p* < 0.01), there were no significant differences between the groups (*p* = 0.86).

**Figure 5:**
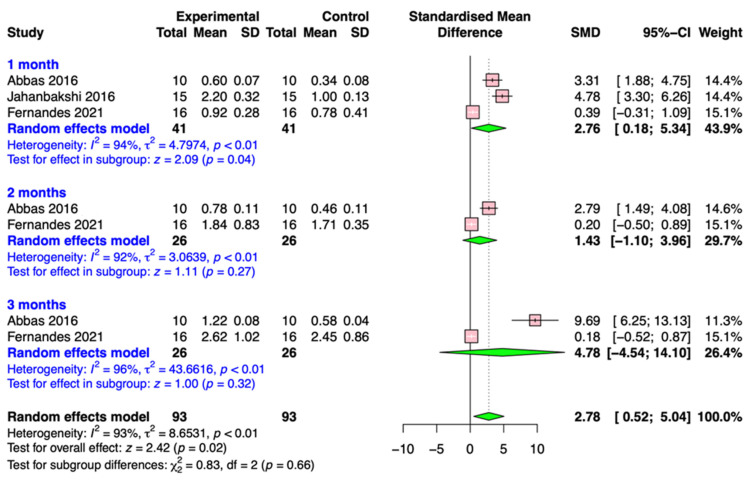
Forest plot of the meta-analysis on studies involving corticotomy [28,30,33].

Three included articles (Abbas et al., 2016 [28]; Jahanbakshi et al., 2016 [30]; Fernandes et al., 2021 [33]) assessed the effect of corticotomy on canine retraction; the aggregated results are presented in Figure 5. The results of the meta-analysis revealed pooled SMD values of accumulative movement distances for corticotomy 2.76 (95% CI, 0.18 to 5.34; *p* = 0.04), 1.43 (95% CI, −1.10 to 3.96; *p* = 0.27), and 4.78 (95% CI, −4.54 to 14.10; *p* = 0.32). Although the test for the overall effect was significant (*p* = 0.02), there were no significant differences between the groups (*p* = 0.66). 

### 3.4. Comparison between Piezocision and Corticotomy

**Figure 6:**
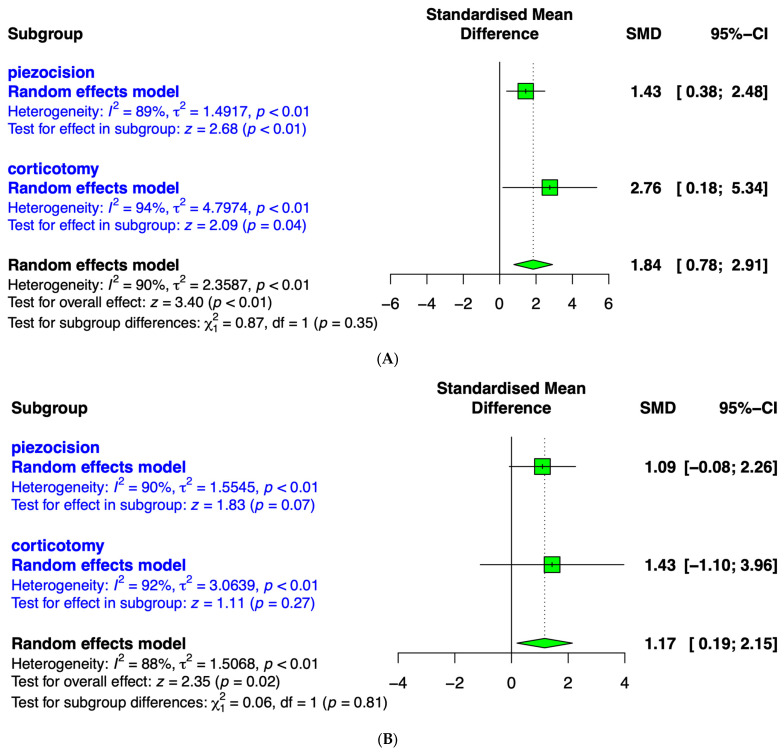

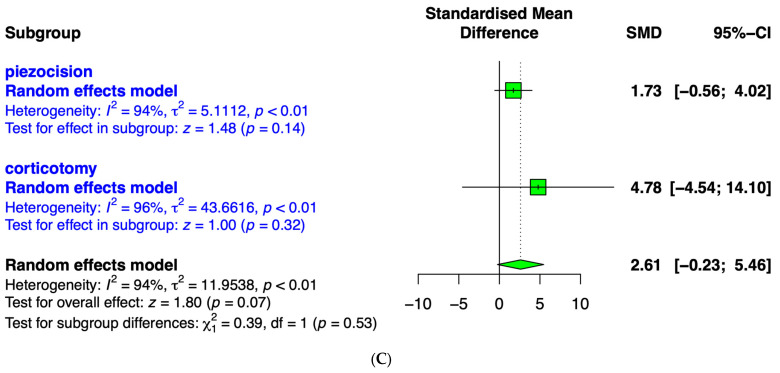
Forest plot illustrating the comparison between piezocision and corticotomy for canine retraction at different time points. (**A**) After 1 month, (**B**) after 2 months, and (**C**) after 3 months of the procedure.

A subgroup analysis was used due to the heterogeneity in surgical methods of the included studies. The literature was divided into two subgroups: flapless piezocision and corticotomy. The results are shown in Figure 6. Considering the *I*^2^ values (1 month = 90%, 2 months = 88%, and 3 months = 94%) and the results of the sensitivity analysis, which indicated significant heterogeneity among the studies, a random effects model was applied. In this study, considering the substantial heterogeneity among the studies, the between-study variance was not assumed to be equal in the meta-analysis of variance.

### 3.5. Canine Retraction after 1 Month

Seven included articles (Abbas et al., 2016 [28]; Aksakalli et al., 2016 [29]; Jahanbakshi et al., 2016 [30]; Alfawal et al., 2018 [18]; Raj et al., 2020 [31]; Alqadasi et al., 2021 [32]; Fernandes et al., 2021 [33]) assessed the effects of flapless piezocision and corticotomy on canine retraction after 1 month (Figure 6A). The meta-analysis results revealed a pooled SMD of accumulative movement distances for both surgical interventions of 1.84 (95% CI, 0.78 to 2.91; *p* < 0.01). When comparing the overall effect sizes of flapless piezocision (1.43; 95% CI, 0.38 to 2.48) and corticotomy (2.76; 95% CI, 0.18 to 5.34), the corticotomy group demonstrated a relatively larger effect size and a wider 95% confidence interval. Although the overall effect was significant (*p* < 0.01), there were no significant differences between the groups (*p* = 0.35).

### 3.6. Canine Retraction after 2 Months

Five included articles (Abbas et al., 2016 [28]; Aksakalli et al., 2016 [29]; Raj et al., 2020 [31]; Alqadasi et al., 2021 [32]; Fernandes et al., 2021 [33]) assessed the effects of flapless piezocision and corticotomy on canine retraction after 2 months (Figure 6B). The meta-analysis results showed a pooled SMD of 1.17 (95% CI, 0.19 to 2.15; *p* < 0.01) for accumulative movement distances for the overall surgical interventions. When comparing the overall effect sizes of flapless piezocision (1.09; 95% CI, −0.08 to 2.26) and corticotomy (1.43; 95% CI, −0.10 to 3.96), the corticotomy group demonstrated a relatively larger effect size and a wider 95% confidence interval. Although the overall effect of treatment was significant (*p* < 0.01), there were no significant differences between the subgroups (*p* = 0.81).

### 3.7. Canine Retraction after 3 Months

Four included articles (Abbas et al., 2016 [28]; Raj et al., 2020 [31]; Alqadasi et al., 2021 [32]; Fernandes et al., 2021 [33]) assessed the effect of flapless piezocision and corticotomy on canine retraction after 3 months (Figure 6C). The results revealed a pooled SMD of 2.61 (95% CI, −0.23 to 5.46; *p* < 0.01) for accumulative movement distances of the surgical interventions. When comparing the overall effect sizes of flapless piezocision (1.73; 95% CI, −0.56 to 4.02) and corticotomy (4.78; 95% CI, −4.54 to 14.10), the corticotomy group demonstrated a relatively larger effect size and a wider 95% confidence interval. Although the overall treatment effect was significant (*p* < 0.01), there were no significant differences between the groups (*p* = 0.53).

## 4. Discussion

In this study, a meta-analysis was conducted to compare the impacts of corticotomy and flapless piezocision on tooth movement. The study determined that both conventional corticotomy and piezosurgery are effective as adjuncts to orthodontic treatment. Moreover, no significant differences were observed in the short-term effectiveness of canine retraction acceleration between the procedures. However, this study is limited by the small number of articles analyzed, and this should be taken into account when interpreting the findings. Both traditional corticotomy and flapless cortico-alveolar perforations serve as effective supplementary surgical techniques for expediting canine retraction, with traditional corticotomy resulting in a 59.85% acceleration and flapless cortico-alveolar perforations, achieving a 44% acceleration compared to conventional retraction after 1 month [34]. A previous report showed that the average rate of canine retraction was 0.65 mm per month for surgical adjunctive procedures [35]. Corticotomy led to increased canine angulation, decreased canine and premolar rotation, and increased molar rotation compared to flap elevation, but these differences were not statistically significant [36]. Another prior investigation examined clinical and radiographic comparisons of changes in bone density and differences in retraction time between buccal and palatal corticotomy performed with a surgical bur, demonstrating no significant differences between the two groups [37].

Corticotomy involves removal of the hard, cortical bone imparting strong resistance to orthodontic forces within the jaw, while preserving the marrow bone to ensure proper blood circulation and uninterrupted integrity of bone tissues to reduce the risk of necrosis and promote tooth movement [13]. In contrast to conventional orthodontic surgery, piezocision is characterized by its minimal invasiveness, featuring small incisions and causing less trauma to the adjacent tissues, ultimately resulting in faster recovery and reduced post-operative discomfort [38]. Thus, orthodontic treatment aided by corticotomy and combined with hard tissue augmentation offers the potential for expeditious tooth repositioning and supplementary advantages such as altering the periodontal phenotype, preserving or enhancing facial bone density, and broadening the range of safe tooth adjustments for patients [39]. Adult patients, particularly those with mandibular anterior teeth displaying dehiscence or fenestration, may benefit from corticotomy-assisted orthodontic treatment for preserving periodontal health, especially in cases of alveolar protrusion [40]. Similarly, utilizing augmented corticotomy during presurgical orthodontic treatment for patients with high-angle skeletal class III malocclusions effectively addresses and safeguards against alveolar bone fenestration and dehiscence around the anterior teeth in alveolar bone defect cases [41]. A preorthodontic augmented corticotomy treatment strategy appeared to effectively prevent alveolar dehiscence and gingival recession around buccally inclined mandibular anterior teeth [42].

It is also important to consider soft tissue management in surgically assisted orthodontics. A prior study indicated that corticotomy-assisted orthodontic treatment combined with soft tissue augmentation resulted in an increase in keratinized tissue width [39]. Moreover, a group that received piezocision showed an increase in the width of keratinized gingiva compared with a group that did not undergo piezocision for molar protraction [43]. A previous report revealed that patients who underwent autogenous gingival grafts in the recession area before orthodontic treatment exhibited a significant reduction in gingival recession after orthodontic treatment compared to before treatment [44]. Also, gingival grafting prior to orthodontics did not lead to an additional reduction in post-orthodontic gingival recession in cases involving retrusion of mandibular incisors [44].

The concept of surgical acceleration emerged following the discovery of the regional acceleratory phenomenon, characterized by an acceleration in normal cellular processes [45,46]. The use of alveolar decortication in conjunction with tooth movement resulted in a notable reduction in both bone volume and bone mineral content [47]. In an animal model of corticotomy-assisted orthodontic tooth movement, there was an elevation in the expression of osteoblast-related cytokines such as osteopontin, bone sialoprotein, and osteocalcin, as well as regulators of osteoclasts like macrophage colony-stimulating factor and activator of nuclear factor kappa B receptor ligand [48]. Similarly, enhanced osteoclast activity was observed on the compression side, whereas osteogenic markers were detected on the tension side during tooth movement with piezocision [49,50]. The concentration of osteocalcin in the gingival crevicular fluid on the tension side of the piezocision group and the level of type I collagen cross-linked C-terminal telopeptide on the compression side of the piezocision group were both increased compared to the corresponding sides in the control group [49].

A prior study suggested that surgically accelerated orthodontic methods did not produce notable negative impacts on the periodontium, root length, or tooth vitality [51]. Another report demonstrated similar adverse effects associated with both surgical and non-surgical interventions [52]. It was reported that piezocision has no adverse impact on the periodontium and offers benefits [43]. To compare post-treatment periodontal status, the accelerated minimally invasive corticotomy-assisted method for treating palatally impacted canines and adjacent teeth showed no short-term impairment in periodontal health compared to the conventional traction method, with both approaches resulting in acceptable post-treatment periodontal outcomes indicated by low gingival index levels [48]. Flapless corticotomy resulted in significantly fewer negative patient-reported outcomes compared to traditional corticotomy, which was associated with mild to moderate levels of pain, swallowing difficulty, discomfort, chewing difficulty, and jaw movement limitation 24 h post-surgery [53]. A previous prospective split-mouth clinical study aimed to compare apical root resorption in anterior teeth between the two corticotomy methods of indentation and vertical techniques, with indentation corticotomy being considered a safer, more effective, minimally invasive, technique-sensitive approach associated with better regional acceleration and rapid healing [54]. Among 14 patients in two previous studies, five displayed noticeable iatrogenic root damage linked to the piezocision procedure [55,56]. When dealing with youth orthodontic patients, it is preferable to opt for a less invasive procedure [57]. Performance of piezocision does require expertise, as indicated in a prior study where the piezocision procedure was exclusively performed by a highly experienced periodontist with over a decade of clinical practice [58]. Computed tomography has been integrated with the piezocision method to allow for computer-aided design, and computer-aided surgical guides aim at averting inadvertent harm by enabling real-time tracking of the piezoelectric instruments during the surgical process [59]. The performance of high-quality clinical trials with reduced bias potential is recommended to draw dependable conclusions about the adverse consequences of the surgical procedures used to expedite orthodontic treatment in individuals of various age groups [51]. 

## 5. Conclusions

Based on the existing literature, both flapless piezocision and conventional corticotomy are effective in accelerating tooth movement when used as adjuncts to orthodontic treatment. No significant difference was observed in the short-term effectiveness of canine retraction acceleration between flapless piezocision and corticotomy. While piezocision may be a favorable option for orthodontic treatment, corticotomy can be considered in cases where additional procedures such as bone grafting are required.

## Figures and Tables

**Table 1:** Characteristics of the included studies.

Study	Region	Study Design	Comparison	Sample Size, n; Sex; Age	Type and Site of Intervention	Duration	Outcome
Abbas 2016 [28]	Egypt	RCT, multi-arm	Corticotomy + OT vs. piezocision + OT; split mouth	20; not given; 15 to 25	Piezocision (upper canines); piezocision (full cortical depth and canine root length) mesial and distal to canine root, 150 *g* force; suture incisions; surgical instrument: piezotome (VarioSurg3; NSK)	3 mo	RCM, molar anchorage loss, canine rotation and inclination, canine root resorption, periodontal parameter
Aksakalli 2016 [29]	Turkey	RCT	Piezocision + OT vs. OT; split mouth	20; f6, m4; 16.3	Piezocision (upper canines); piezocision (3 mm depth and 10 mm length) mesial and distal to canine, 150 *g* force; surgical instrument: piezosurgery knife (BS1)	Until class I canine relationship	Compare the extent of distalization and transversal changes, gingival indices, mobility scores
Jahanbakhshi 2016 [30]	Iran	RCT	Buccal corticotomy + OT vs. OT; split mouth	15; f15; 25	Buccal corticotomy (distal to canine, mesial to U5, vertical groove length 1 mm × depth 0.5~1 mm, 10 spherical perforation with round 2 bur); 200 g force, simple vertical loop	4 mo	Compare average velocity of tooth movement (monthly and total)
Alfawal 2018 [18]	Syria	RCT, multi-arm	Piezocision + OT vs. laser-assisted flapless corticotomy + OT; split mouth	36; f24, m12; 18.08	Piezocision (upper canines); 2 corticotomies (3 mm in depth and 10 mm in length) in the buccal at equal distance from the upper canine and second premolar, 150 *g* force; surgical instrument: piezosurgery knife (BS1); LAFC (3 mm in depth; 8 mm in length) at equal distance from the upper canine and second premolar, 150 *g* force; surgical instrument: ER:YAG laser, 200 mJ, 12 Hz, 3 W	4 mo	RCM, molar anchorage loss
Raj 2020 [31]	India	RCT	Piezocision + OT vs. OT Split mouth	20; f14, m6; 20–25	Piezocision (upper canine); piezocision (3 mm depth × 10 mm length); surgical instrument: piezosurgery knife (Acteon, BS1); 150 *g* Force canine retraction	6 mo	Compare rate of canine extraction, resultant alveolar bone level, root resorption, and periodontal parameter
Alqadasi 2021 [32]	China	RCT, paralle-group	Piezoincision vs. convetional; split mouth Micro-osteo perforation vs. convetional; split mouth Piezoincision vs. MOP	21; f12, m9; 15 to 40	MOPs (flapless perforation using an automated mini-implant driver, 1.5–2 mm in diameter, 5–7 mm in depth) Piezoincision using piezoelectric instruments (Surgybone; Silfradent) (2 mm below the crest of the alveolar ridge and 3 mm in length and 3–5 mm in depth)	3 mo	Rate of tooth movement, Root resorption, bone height
Fernandes 2021 [33]	Brazil	RCT	Corticitomy + OT vs. piezocision + OT vs. Corticotomy + piezocision; Split mouth	47; m19, f28; 15~38	Piezocision (upper canine) (3 mm depth × 5 mm length), (Acteon, BS1); Corticotomy (Vertical groove: mesial and distal of canine root, mesial of second premolar/horizontal groove: above canine apex/spherical cortical perforation with round 2 bur, canine to mesial of second premolar); 1.15 N force canine retraction	6 mo	Compare cumulative distal movement of canines and molecular analysis of gingival crevicular fluid

RCT: randomized clinical trial; OT: orthodontic treatment; MOP: micro-osteo perforation; RCM: rate of canine movement; mo: months.

## Data Availability

This article contains all of the information that was created or examined during this investigation.

## Supplementary Materials

The following supporting information can be downloaded at https://www.mdpi.com/article/10.3390/medicina59101804/s1. Table S1: Search strategy of the online databases; Table S2: Excluded studies from full-text reading; Table S3: Risk of bias. References [60,61,62,63,64,65,66,67,68,69] are cited in Table S2.
