# Supplementary material for: Comparative Efficacy of Traditional Corticotomy and Flapless Piezotomy in Facilitating Orthodontic Tooth Movement: A Systematic Review and Meta-Analysis
Source: Medicina (Kaunas). 2023 Oct 10;59(10):1804. doi: 10.3390/medicina59101804 (PMC10608606; doi:10.3390/medicina59101804)
Supplement: Supplementary file 1 [file medicina-59-01804-s001.zip › medicina-2629393-supplementary.pdf]

Supplementary Table S1: Search strategy of the online databases.

DB	Search	Search strategy
**Medline via PubMed**	#1	"Orthodontics"[Mesh Terms]
	#2	"Orthodontic treatment"[Text Word] OR "Orthodontic"[Text Word]
	#3	"Tooth Movement Techniques"[MeSH Terms]
	#4	"Tooth Movement"[Text Word]
	**#5 Combine**	**#1 OR #2 OR #3 OR #4**
	#6	"corticotomy"[Text Word] OR "corticotomy assisted"[Text Word] OR "corticotomy-facilitated"[Text Word] OR "surgical-facilitated"[Text Word] OR "wilckodontics"[Text Word] OR "accelerated osteogenic"[Text Word] OR "periodontally accelerated osteogenic"[Text Word] OR "decortication"[Text Word] OR "decorticated"[Text Word] OR "augmented corticotomy"[Text Word] OR (("corticotomies"[All Fields] OR "corticotomy"[All Fields]) AND "augmentation"[Text Word]) OR (("corticotomies"[All Fields] OR "corticotomy"[All Fields]) AND "bone graft"[Text Word])
	#7	"piezosurgery"[MeSH Terms]
	#8	"piezosurgeries"[Text Word] OR "piezo-electric surgery"[Text Word] OR "piezo eletric surgery"[Text Word] OR "piezo-electric surgeries"[Text Word] OR "surgeries, piezo-electric"[Text Word] OR "surgery, piezo-electric"[Text Word] OR "piezo-electric bone surgery"[Text Word] OR "bone surgeries, piezo-electric"[Text Word] OR "bone surgery, piezo-electric"[Text Word] OR "piezo electric bone surgery"[Text Word] OR "piezo-electric bone surgeries"[Text Word] OR "surgeries, piezo-electric bone"[Text Word] OR "surgery, piezo-electric bone"[Text Word] OR "piezocision"[Text Word] OR "piezosurgical"[Text Word] OR "piezosurgical, corticotomy"[Text Word] OR "piezosurgical, decortication"[Text Word] OR "piezosurgical, corticotomies"[Text Word]
	**#9 Combine**	**#6 OR #7 OR #8**
	**#10 Combine**	**#5 AND #9**
	**#11 Limit**	**#10 AND (randomizedcontrolledtrial[Filter])**
**Cochrane library**	#1	MeSH descriptor: [Orthodontics] explode all trees
	#2	orthodontic treatment OR orthodontic
	#3	MeSH descriptor: [Tooth Movement Techniques] explode all trees
	#4	tooth movement
	**#5 Combine**	**#1 OR #2 OR #3 OR #4**

	#6	corticotomy OR corticotomy assisted OR corticotomy-facilitated OR surgical-facilitate OR wilckodontics OR accelerated osteogenic OR periodontally accelerated osteogenic OR decortication OR decorticated OR augmented corticotomy OR corticotomies OR corticotomy, augmentation OR corticotomies, augmentation OR corticotomy, bone graft OR corticotomies, bone graft
	#7	MeSH descriptor: [Piezosurgery] explode all trees
	#8	piezosurgeries OR piezo-electric surgery OR piezo electric surgery OR piezo-electric surgeries OR surgeries, piezo-electric OR surgery, piezo-electric OR piezo-electric bone surgery OR bone surgeries, piezo-electric OR bone surgery, piezo-electric OR piezo electric bone surgery OR piezo-electric surgeries OR surgeries, piezo-electric bone OR surgery, piezo-electric bone OR piezocision OR piezosurgical OR piezosurgical, corticotomy OR piezosurgical, decortication OR piezosurgical, corticotomies
	**#9 Combine**	**#6 OR #7 OR #8**
	**#10 Combine**	**#5 AND #9**
	**#11**	**Cochrane Reviews / Trials**
**Embase**	#1	orthodontics'/exp OR orthodontics
	#2	orthodontic
	#3	tooth movement'
	#4	orthodontic tooth movement'
	**#5 Combine**	**#1 OR #2 OR #3 OR #4**
	#6	corticotomy' OR 'corticotomy assisted' OR 'corticotomy-facilitated' OR 'surgical-facilitate' OR 'wilckodontics' OR 'accelerated osteogenic' OR 'periodontally accelerated osteogenic' OR 'decortication' OR 'decorticated' OR 'augmented corticotomy' OR 'corticotomies' OR 'corticotomy, augmentation' OR 'corticotomies, augmentation' OR 'corticotomy, bone graft' OR 'corticotomies, bone graft'
	#7	piezosurgery'
	#8	piezosurgeries' OR 'piezo-electric surgery' OR 'piezo electric surgery' OR 'piezo-electric surgeries' OR 'surgeries, piezo-electric' OR 'surgery, piezo-electric' OR 'piezo-electric bone surgery' OR 'bone surgeries, piezo-electric' OR 'bone surgery, piezo-electric' OR 'piezo electric bone surgery' OR 'piezo-electric surgeries' OR 'surgeries, piezo-electric bone' OR 'surgery, piezo-electric bone' OR 'piezocision' OR 'piezosurgical' OR 'piezosurgical, corticotomy' OR 'piezosurgical, decortication' OR 'piezosurgical, corticotomies'
	**#9 Combine**	**#6 OR #7 OR #8**
	**#10 Combine**	**#5 AND #9**

	**#11 Limit**	**#10 AND 'randomized controlled trial'/de**
**OpenGrey** **http://www.opengrey.eu/**	#1	orthodontics
	#2	orthodontic treatment OR orthodontic
	#3	Tooth movement techniques
	#4	Tooth movement
	**#5 Combine**	**#1 OR #2 OR #3 OR #4**
	#6	corticotomy OR corticotomy assisted OR corticotomy-facilitated OR surgical-facilitate OR wilckodontics OR accelerated osteogenic OR periodontally accelerated osteogenic OR decortication OR decorticated OR augmented corticotomy OR corticotomies OR corticotomy, augmentation OR corticotomies, augmentation OR corticotomy, bone graft OR corticotomies, bone graft
	#7	piezosurgery
	#8	piezosurgeries OR piezo-electric surgery OR piezo electric surgery OR piezo-electric surgeries OR surgeries, piezo-electric OR surgery, piezo-electric OR piezo-electric bone surgery OR bone surgeries, piezo-electric OR bone surgery, piezo-electric OR piezo electric bone surgery OR piezo-electric surgeries OR surgeries, piezo-electric bone OR surgery, piezo-electric bone OR piezocision OR piezosurgical OR piezosurgical, corticotomy OR piezosurgical, decortication OR piezosurgical, corticotomies
	**#9 Combine**	**#6 OR #7 OR #8**
	**#10 Combine**	**#5 AND #9**
**PQDT OPEN (from proQuest)** **http://pqdtopen.proquest.com/**	#1	fulltext(orthodontics)
	#2	fulltext(orthodontic treatment) OR fulltext(orthodontic)
	#3	fulltext(Tooth movement techniques)
	#4	fulltext(Tooth movement)
	**#5 Combine**	**#1 OR #2 OR #3 OR #4**
	#6	fulltext(corticotomy OR corticotomy assisted OR corticotomy-facilitated OR surgical-facilitate OR wilckodontics OR accelerated osteogenic OR periodontally accelerated osteogenic OR decortication OR decorticated OR augmented corticotomy OR corticotomies OR corticotomy, augmentation OR corticotomies, augmentation OR corticotomy, bone graft OR corticotomies, bone graft)
	#7	fulltext(piezosurgery)

	#8	fulltext(piezosurgeries OR piezo-electric surgery OR piezo electric surgery OR piezo-electric surgeries OR surgeries, piezo-electric OR surgery, piezo-electric OR piezo-electric bone surgery OR bone surgeries, piezo-electric OR bone surgery, piezo-electric OR piezo electric bone surgery OR piezo-electric surgeries OR surgeries, piezo-electric bone OR surgery, piezo-electric bone OR piezocision OR piezosurgical OR piezosurgical, corticotomy OR piezosurgical, decortication OR piezosurgical, corticotomies)
	**#9 Combine**	**#6 OR #7 OR #8**
	**#10 Combine**	**#5 AND #9**

Supplementary Table S2: Excluded studies from full-text reading.

Reasons for exclusion	References
**Not found**	60. Simre S.S, Rajanikanth K. Evaluation of conventional corticotomy with novel piezosurgery in orthodontic treatment – Study protocol for a comparative study. Eur J of Mol Clin Med 2020;7(2):2128–2131.
**Not randomized trials**	61. Deepak C. Corticotomy assisted fixed orthodontic treatment vs non corticotomy assisted fixed orthodontic treatment: A randomised clinical trial study. Indian J Public Health Res Dev 2019;10(1):2873-2875.
**Questionable data source**	62. Sonone T.P, Nawab A, Krishnaraj P, et al. The effects of corticotomy. and piezocision in orthodontic canine retraction: A randomized controll ed clinical trial. J Pharm Bioallied Sci 2022;14(5):S757–S764.
**Outcome measured at different time points**	63. Al-A'athal H.S, Al-Nimri K, Alhammadi M.S. Analysis of canine retraction and anchorage loss in different facial types with and without. piezocision a split-mouth-design, randomized clinical trial. Angle Orthod 2022;92(6):746-754.<br>64. Hawkins V.M, Papadopoulou A.K, Wong M, et al. The effect of piezocision vs no. piezocision on maxillary extraction space closure: A split-mouth, randomized controlled clinical trial. Am J Orthod Dentofacial Orthop 2022;161(1):7-19.
**No control group**	65. Farid K.A, Eid A.A, Kaddah A, et al. The effect of combined corticotomy and low level laser therapy on the rate of orthodontic too th movement: Split mouth randomized clinical trial. Laser Therapy 20 19;28(4):275-283.<br>66. Alfawal A.M.H, Hajeer M.Y, Ajaj M.A, et al. Evaluation of patient -centered outcomes associated with the acceleration of canine retraction. by using minimally invasive surgical procedures: A randomized clinical controlled trial. Dent Med Probl 2020;57(3):285-293.<br>67. Türker G, Yavuz I, Gönen B. Which method is more effective for accel erating canine distalization short term, low-level laser therapy or piezoc ision? A split-mouth study. J Orofac Orthop 2021;82(4):236-245.<br>68. Simre S.S, Rajanikanth K, Bhola N, et al. Comparative assessment of. corticotomy facilitated rapid canine retraction using piezo versus bur: A randomized clinical study. J Oral Biol Craniofac Res 2022;12(1):182

	-186.
**No research article**	69. Qabool H, Sukhia R.H. Assessment of rate of tooth movement and duration of canine retraction using piezocision. Am J Orthod Dentofacial Orthop 2022;161(6):766.

Supplementary Table S3: Risk of bias

**Study**	**D1: Randomisation process**	**D2: Deviations from the intended interventions**	**D3: Missing outcome data**	**D4: Measurement of the outcome**	**D5: Selection of the reported result**
Abbas 2016 [28]	Low risk: "The randomization was performed with coin tosses to prevent selection bias. The sides contralateral to the procedures served as the controls in both groups." (Page 474).	Low risk: No deviation From the Intended intervention has been reported.	Low risk: No dropouts were reported.	Low risk: "The anteroposterior crown tip movements of the canines were assessed at 6 time points with the method described by Ziegler and Ingervall." (Page 476).	Some concerns: No information on whether the research results were analyzed before the unblinding of the intervention outcome data.
Aksakalli 2016 [29]	High risk: "The study involved a split-mouth	Low risk: No deviation From the	Low risk: No dropouts were	Low risk: "Pre- and postdistalization model casts	Some concerns: No information

	design, with the experimental quadrant selected by randomization." (Page 60).<br><br>The description of the randomization is not specific, and it's impossible to conceal the allocation of intervention.	Intended intervention has been reported.	reported.	were scanned using the 3Shape R900 scanner. The models were superimposed, and the changes in the models were evaluated for canine distalization changes. The superimpositions were performed by selecting the medial end of the third palatal rugae as reference points." (Page 60).	on whether the research results were analyzed before the unblinding of the intervention outcome data.
Jahanbakhshi 2016 [30]	High risk:<br><br>"This study was performed by using split mouth design method. In a randomized manner, one side of the maxillary arch on which corticotomy	Low risk:<br><br>No deviation<br><br>From the<br><br>Intended intervention has been reported.	Low risk:<br><br>No dropouts were<br><br>reported.	High risk:<br><br>"Using a compass and a caliper, the distance between the canine and second premolar was measured on each side, and the traveled distance was	Some concerns:<br><br>No information on whether the research results were analyzed before the unblinding of the intervention outcome

	was applied was considered as the experimental group," (Page 305).<br><br>The description of the randomization is not specific, and it's impossible to conceal the allocation of intervention.			divided by the time of treatment, to specify the rate of tooth movement." (Page 305).	data.
Alfawal<br><br>2018 [18]	Low risk:<br><br>"Simple randomization was conducted by one of the academic stuff at the Department of Orthodontics using computer-generated random numbers with an allocation ratio of 1:1. Allocation	Low risk:<br><br>No deviation<br><br>From the<br><br>Intended intervention has been reported.	Low risk:<br><br>No dropouts were<br><br>reported.	Low risk:<br><br>"Maxillary casts were photographed digitally with focal projection vertical to the occlusal plane for the correction of magnification regarding the linear measurements. The measurements were carried out on the digital	Low risk:<br><br>"Blinding of personnel and participants were not applicable. Therefore, blinding was applied only for outcomes' assessor." (Page 3).

	sequence was conclealed using sequentially numbered, opaque, sealed envelopes, which were opened only after the completion of leveling and alignment stage of the dental arches.” (Page 2).			photographs using AudaxCeph version 3.4.2.2710 with the method described by Ziegler and Ingervall.” (Page 4).	

Raj 2020 [31]	Low risk: "Computer-generated randomization and quadrant allocation was carried out." (Page e20).	Some concerns: "One patient did not receive allocation intervention due to lack of consent." (Fig 5)(Page e23).	Low risk: No dropouts were reported.	Low risk: "For standardization of these measurements, a customized acrylic stent was fabricated at the first visit and was sectioned and fitted to the canine for subsequent measurements. The rate of canine retraction was measured by estimating the change in the distance between the mesial aspect of the molar tube slot and the distal aspect of the canine bracket, measured intraorally by digital Vernier calipers." (Page e21).	Some concens: No information on whether the research results were analyzed before the unblinding of the intervention outcome data.

Alqadasi 2021 [32]	Low risk: “The participants and sides were randomly allocated using a computer-generated random list simple method (https://www.random.org/).” (Page 337).	Low risk: No deviation From the Intended intervention has been reported.	Low risk: No dropouts were reported.	Low risk: “The investigators performing the measurements and data analysis were blinded from the group assignments.” (Page 640), it was possibly done.	Low risk: “Blinding was possible during the measurement stage and was accomplished using digital coding of the CBCT and the digital models. Decoding was performed before submitting the data for statistical analysis.” (Page 337).

Fernandes 2021 [33]	Low risk: “Prior to the start of the study, randomization by block was performed by Quick Clacs in a randomization center, by a person not associated to the patients’ recruitment center. Recruitment center individuals, including orthodontist and oral surgeon, did not have access to the allocation sequence, ensuring the concealment of allocation.” (Page 6).	Some concerns: “There were 4 patient dropouts: one, before allocation gave up any orthodontic treatment, and the other 3 moved to different cities and were unable to proceed with treatment.” (Page 7).	Low risk: No dropouts were reported.	Low risk: “Measurements were performed using the digital model obtained on T0, using the OrthoAnalyser 2015 software. The other digital models were superimposed with this reference model by matching the palatal rugae, since this structure is stable even during OTM.” (Page 4).	Low risk: “For the digital models’ measurement, the operator was not aware of the patients group, as well as whether the side was experimental or control, Digital models were previously coded, and they were also cut up to 1 or 2 mm above the teeth cervical margin in the buccal side in order to prevent visualization of possible surgical scars.” (Page 6).
